# Supplementary material for: A Novel Estrogen Receptor β Agonist Diminishes Ovarian Cancer Stem Cells via Suppressing the Epithelial-to-Mesenchymal Transition
Source: Cancers (Basel). 2022 May 6;14(9):2311. doi: 10.3390/cancers14092311 (PMC9105687; doi:10.3390/cancers14092311)
Supplement: Supplementary file 1 [file cancers-14-02311-s001.zip › cancers-1695563-supplementary.pdf]

# A novel estrogen receptor β agonist depletes ovarian cancer stem cells via suppressing epithelial-to-mesenchymal transition

Ananya Banerjee et al.

## Supplemental Figures

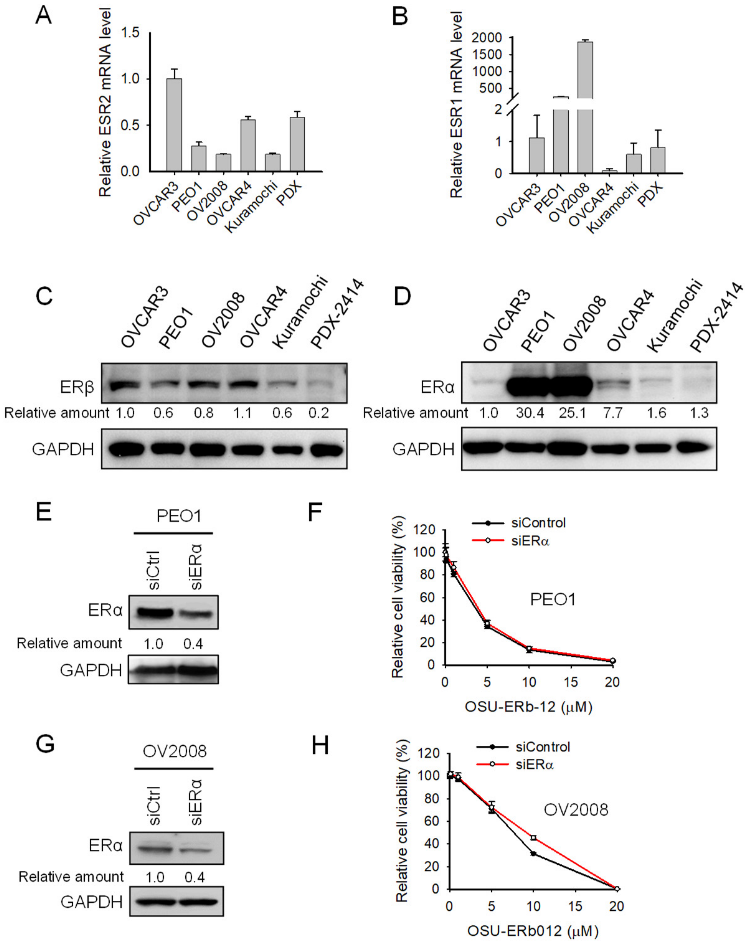


**Figure S1.** ERα and ERβ expression in ovarian cancer cells. A-D. Expression of ERα and ERβ at both the mRNA level (A, B) and protein level (C, D) in a panel of ovarian cancer cell lines and a PDX tissue was determined using qRT-PCR and immunoblotting, respectively. Relative amount of Erβ and ERα were quantified relative to GAPDH and normalized by OVCAR3 cells. E-H. PEO1 and OV2008 cells were transfected with either siCtrl or siERα for 48 h, treated with OSU-ERb-12 for 7 days. The ERα protein level was determined using immunoblotting (E, G); cell viability was determined using the methylene blue assay (F, H).

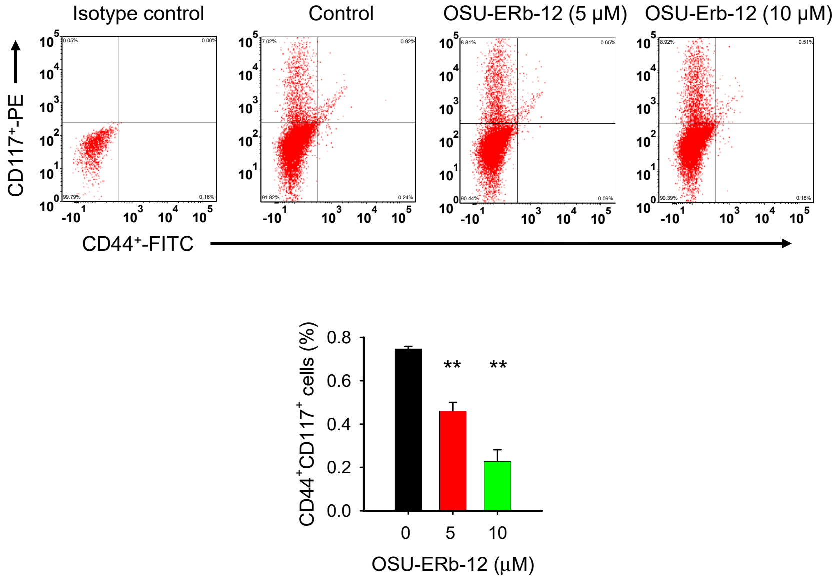


**Figure S2.** ERβ agonist OSU-ERb-12 reduces the CSC population characterized by $CD44^{+}CD117^{+}$ in OVCAR3 cells. OVCAR3 cells were treated with OSU-ERb-12 for 72 h. The abundance of $CD44^{+}CD117^{+}$ cells were analyzed using flow cytometry. N=3, bar: SD, **: P<0.01.

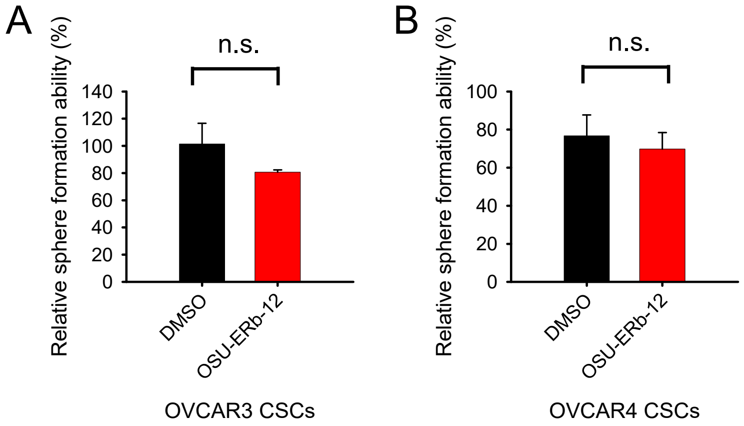


**Figure S3.** ERβ agonist OSU-ERb-12 does not affect the stemness of ovarian CSCs. OVCAR3 (A) and OVCAR4 (B) sphere cells were treated with DMSO or OSU-ERb-12 for 24 h, sphere forming assay was conducted to determine the sphere formation ability. n.s.: No significant.

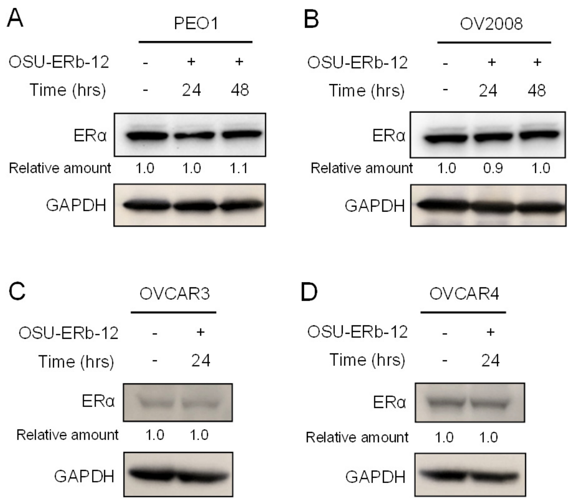


**Figure S4.** ERβ agonist OSU-ERb-12 does not affect the ERα protein level. A panel of ovarian cancer cell lines were treated with OSU-ERb-12 (10 μM) for 24 h or 48 h. Immunoblotting was conducted to determine the protein level of ERα. GAPDH was also determined to serve as a loading control. The band intensity of the ERα was quantitated and normalized by that of GAPDH. The relative protein amount was further calculated by comparing to the corresponding control group.

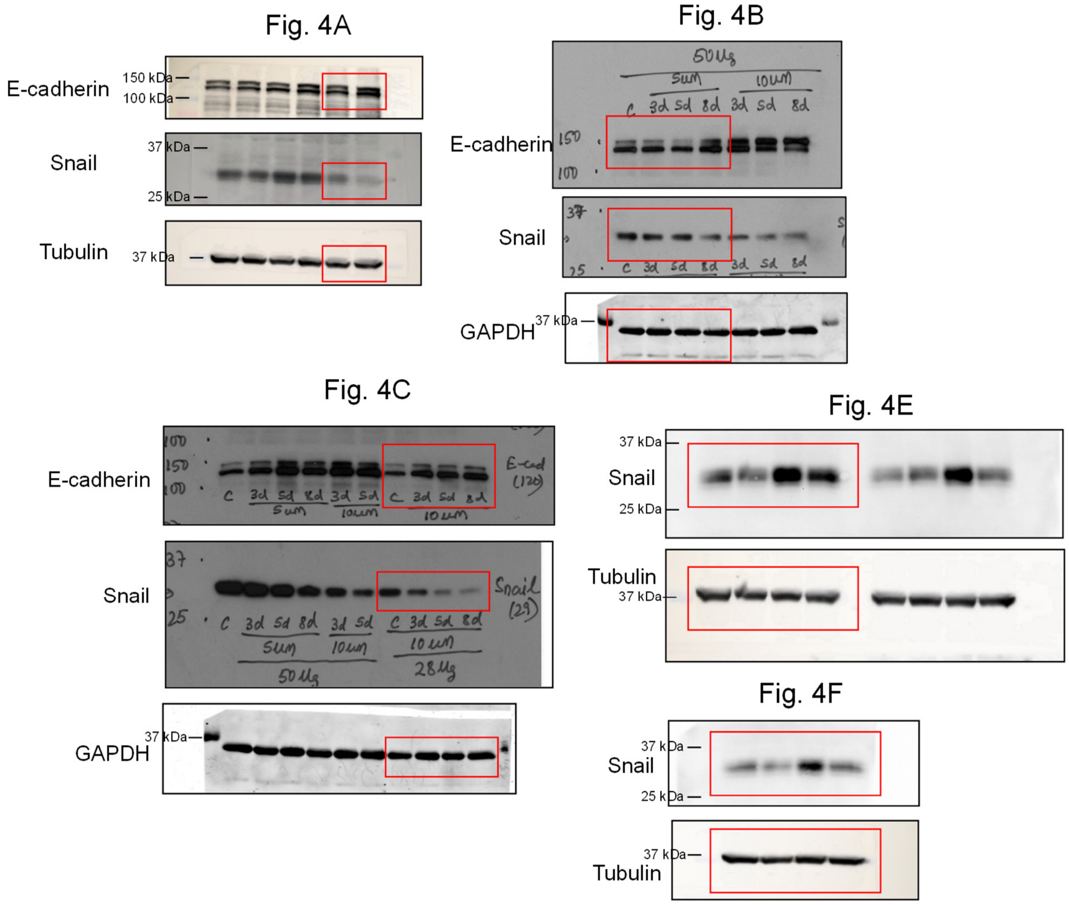


**Figure S5.** Uncropped Western blots of Figure 4.

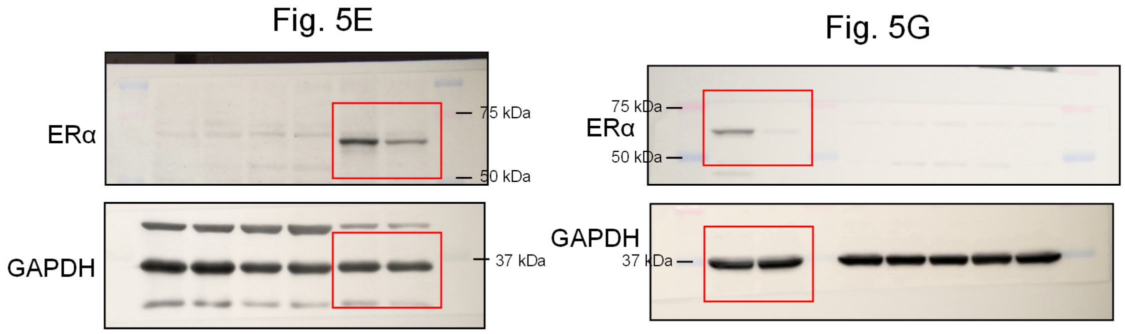


**Figure S6.** Uncropped Western blots of Figure 5.

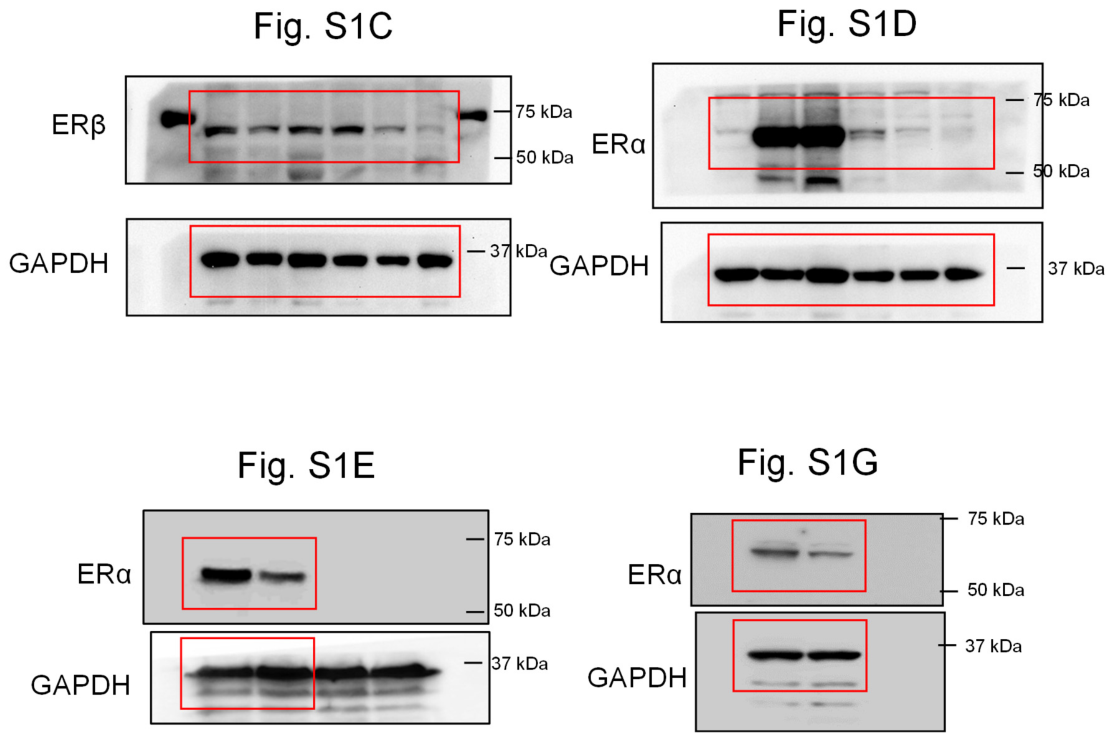


**Figure S7.** Uncropped Western blots of Figure S1.

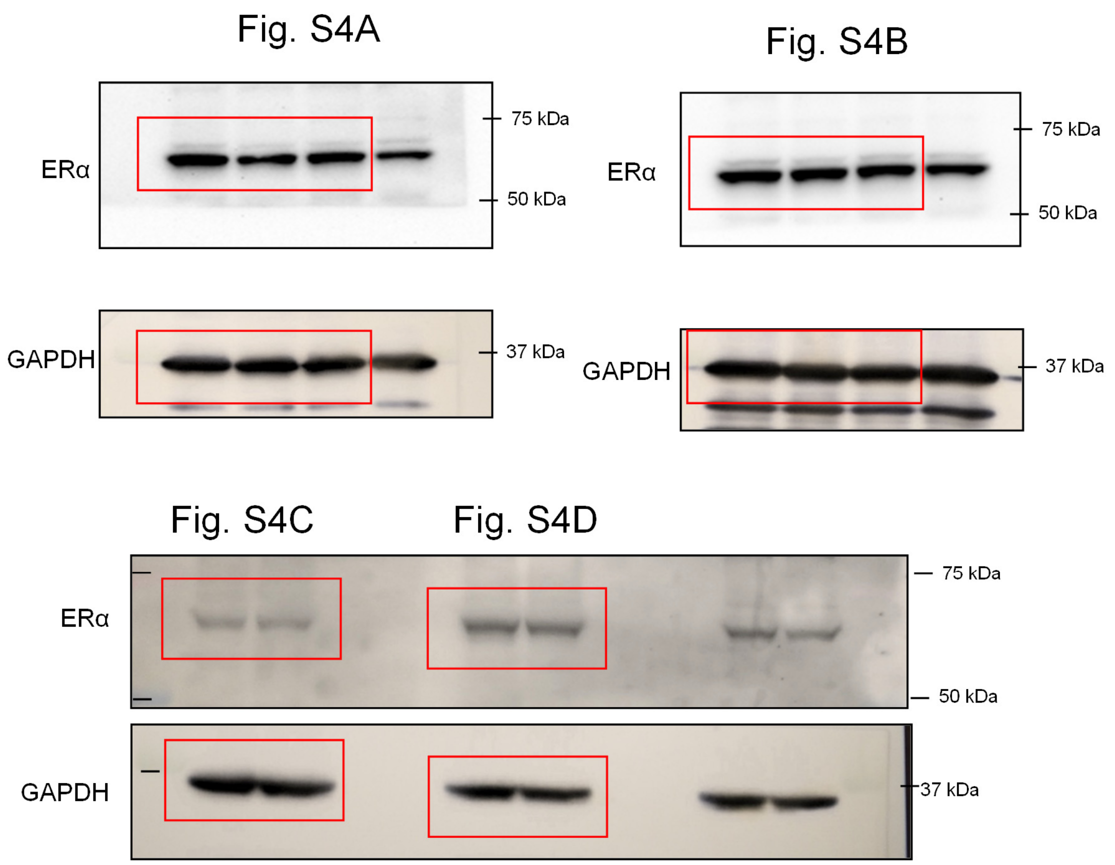


**Figure S8.** Uncropped Western blots of Figure S4.
